# A Comparison of Peripheral Blood Smears, Autologous Cell Cultures, and Reverse Line Blot Hybridisation in Screening for *Anaplasma*/*Ehrlichia* in Roaming Dogs and Symptomatic Dogs in Trinidad

**DOI:** 10.3390/pathogens10111431

**Published:** 2021-11-04

**Authors:** Karla Georges, Chuckwudozi Ezeokoli, Godwin Isitor, Alex Mutani, Olivier Sparagano, Candice Sant

**Affiliations:** 1 School of Veterinary Medicine, Faculty of Medical Sciences, University of the West Indies, St. Augustine, Trinidad and Tobago; isitorgn@gmail.com (G.I.); amutani@gmail.com (A.M.); Candice.Sant@sta.uwi.edu (C.S.); 2 College of Veterinary Medicine, Federal University of Agriculture, Makurdi 970231, Benue State, Nigeria; ezeokoli01@yahoo.com; 3 Jockey Club College of Veterinary Medicine and Life Sciences, City University of Hong Kong, Kowloon Tong, Kowloon, Hong Kong, China; asparaga@cityu.edu.hk

**Keywords:** cell culture, dogs, *Ehrlichia canis*, polymerase chain reaction, reverse line blot hybridisation, Trinidad

## Abstract

This study compared two methods to detect cases of canine ehrlichiosis in a field setting. One method was a polymerase chain reaction for the 16S rRNA gene followed by reverse line blot hybridisation with genera and species-specific probes for *Anaplasma/Ehrlichia*. The second method was an autologous cell culture of peripheral leucocytes isolated from heparinised blood and maintained in a homologous canine serum in Dulbecco’s Modified Eagle medium without antibiotics. The cultures were examined under light microscopy for inclusion bodies after 48 h. Leucocytes were successfully propagated for 20 of the 34 samples submitted for autologous cell culture. Inclusion bodies were observed after cell culture in leucocytes of eight dogs. Two dogs were positive to the *Anaplasma/Ehrlichia* genera probe and six dogs were positive to the *E. canis* probe after reverse line blot hybridisation. There was acceptable agreement between reverse line blot hybridisation and cell culture results. Both reverse line blot hybridisation and autologous cell cultures can be used to detect *E. canis* in subclinical and clinical cases of disease. A definitive diagnosis of *E. canis* is best achieved by a combination of clinical signs, positive autologous cell culture, and reverse line blot hybridisation results.

## 1. Introduction

Canine monocytic ehrlichiosis (CME) is a tick-transmitted disease caused by members of the family Rickettsia and genus *Ehrlichia*. *Rhipicephalus sanguineus* (the brown dog tick) is the major vector of this haemopathogen worldwide [1]. CME was first recognised in Algeria in 1935 and is now known to occur worldwide [2]. As canine ehrlichiosis is endemic in Trinidad, most veterinarians treat for ehrlichiosis after routine clinical examinations of suspected cases, even though the animals present with non-specific or vague clinical signs. Canine ehrlichiosis has an incubation period of 8–20 days followed by an acute, subclinical, and then chronic phase. However, the differences between the acute and chronic phases may be obscure in areas where the disease is endemic. Treating canine ehrlichiosis in its acute phase is important for the best prognosis [3] and hence a definitive diagnosis is desired. In the absence of a diagnostic test, the complete blood count (CBC) is the preferred initial diagnostic method used by most veterinarians in Trinidad hoping to find either thrombocytopaenia, pancytopaenia or the characteristic morulae in the cytoplasm of mononuclear cells in thin blood smears for a definitive diagnosis. Since initial blood smears may be negative, the response to treatment of either doxycycline hydrochloride (5–7 mg/kg SID for three weeks), imidocarb diproprionate (6.6 mg/kg, subcutaneously, repeated after two weeks), or a combination of the two drugs is often used for a confirmatory diagnosis of canine ehrlichiosis. It is important, therefore, to offer clinicians a rapid definitive diagnosis of *E. canis* to effectively treat affected dogs, eliminate the use of unnecessary medication, and reduce further complications following a late diagnosis. 

A previous study in Trinidad used autologous cell cultures derived from the buffy coat of heparinised canine, equine, bovine, and ovine blood samples. This study detected the presence of *Ehrlichia*-like morulae in these species and concluded that the autologous cell culture technique may be applied for the routine screening of these organisms. The use of autologous cell cultures also eliminates the need for the maintenance of specific cell lines in vitro, which may be time consuming and expensive [4]. 

The aim of this study was to detect *E. canis* in blood using autologous cell cultures and a reverse line blot hybridisation assay (RLB). The sample frame comprised of free roaming dogs and dogs showing clinical signs suggestive of *E. canis* infection on presentation to the veterinary hospitals and clinics. This study would evaluate both techniques for the routine screening of arthropod-transmitted haemopathogens in a clinical setting.

## 2. Results

Twenty-eight samples were obtained from the Humane Society (group 1) and six samples were suspect cases obtained from other veterinarians throughout Trinidad (group 2). Direct microscopy on the ethylenediaminetetraacetic acid (EDTA) blood smears revealed no observable *Anaplasma/Ehrlichia* morulae in any samples; however, the haematological profile of one sample was highly suggestive of *E. canis* because of a pancytopaenia. 

Samples were also tested using RLB. Of the 28 dogs in group 1, six (21.4%) were positive at any probe with three (10.7%) dogs positive at the *E. canis* probe. Three (50.0%) samples in group 2 were positive at the *E. canis* probe.

**Figure 1:**
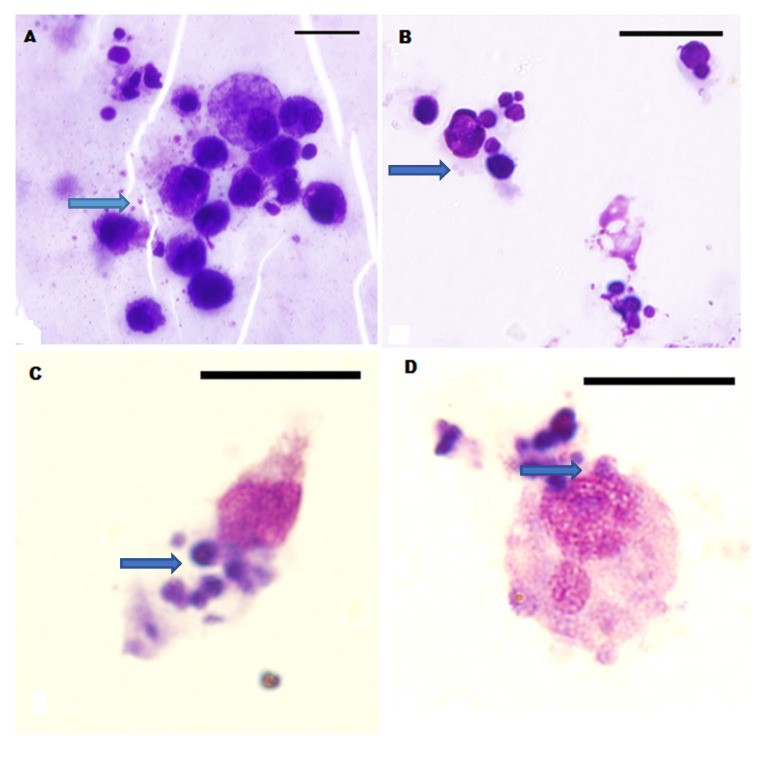
Leucocytes with *E. canis* morulae (blue arrows) in cell culture stained by Wright-Giemsa method (Figure 1). The roughly spherical *E. canis* morulae inclusion bodies abound within cytoplasmic extensions of the leucocytes in Panels (**A**–**D**). Note the enlargement of some of the leucocytes (**C**,**D**) with disrupted portions of the cytoplasm containing morulae. Bar = 13.7 µm.

With regard to the direct microscopy of the autologous cell culture, successful results were obtained for 20/34 (58.8%) samples, with 16/28 (57.1%) and 4/6 (66.7%) from the dogs in groups 1 and 2, respectively. A slide was considered positive for *E. canis* if initial bodies or morulae were observed in the cytoplasm of leucocytes (Figure 1). The *E. canis* morulae inclusion bodies of variable sizes and densities were largely observed within disrupted cytoplasmic extensions of leucocytes. The leucocytes were also of variable sizes, mostly of the mononuclear type, and characterized by a high nuclear-to-cytoplasmic ratio, except for the morulae-infected leucocytes that were mostly enlarged with disrupted cytoplasmic portions laden with the morulae (Figure 1C,D). The morulae were typically clustered in aggregates ranging between two and five, and were deeply basophilic with a more or less even core. They measured between 0.5 and 4 µm in diameter.

The person interpreting the slide after the cell culture was not aware of the clinical signs or RLB results. For dogs in group 1, three out of six positive culture results were negative on RLB, and one out of ten that were negative on culture appeared positive at the *Anaplasma/Ehrlichia* genera probe on RLB. There was a moderate agreement (kappa = 0.43, *p* = 0.07) between culture and RLB results for the dogs sampled in group 1 and perfect agreement (kappa = 1.0, *p* = 0.05) between RLB and culture results for suspect positive cases (group 2). The overall agreement between the RLB and cell culture results was acceptable (kappa = 0.565, *p* = 0.01). These results are displayed in Table 1, Table 2 and Table 3.

Of the six dogs in group 2, five presented with anorexia and three presented with pyrexia. The other signs observed in clinically ill dogs were corneal oedema, splenomegaly, and bleeding. On examination of the dogs in group 1, 19 appeared in good condition. Pyrexia was observed in two dogs and the mean rectal temperature was 38.3 °C.

Ticks were observed on eight dogs at the time of sampling. Fleas were observed on one dog. Six dogs with ticks were negative by both RLB and cell culture, one dog with ticks was positive by both RLB and cell culture, and one dog with ticks was positive by cell culture only. The one dog that had fleas was positive on RLB and cell culture.

The median haematocrit levels for dogs in groups 1 and 2 were 0.42 L/L and 0.31 L/L, respectively. Haematocrit values less than 0.35 L/L were observed for eight dogs; five were from clinically ill dogs (group 2), and three were from group 1. Platelet count data were analysed for 12 and 4 dogs in groups 1 and 2, respectively. Thrombocytopaenia (platelet counts of <200 × 10^9^ L/L) was observed in 5/12 Humane Society dogs (group 1), with one dog being RLB-positive at the *Anaplasma/Ehrlichia* catch-all probe only. Three out of four clinically ill and thrombocytopenic dogs were positive at the *E. canis* probe. Neutrophilia and eosinophilia, either together or separately, were the most common haematological abnormalities observed in this study.

## 3. Discussion

This study compared the diagnostic value of peripheral blood smears, autologous cell cultures, and RLB in detecting *E. canis* in a clinical setting. *E. canis* was not observed in any of the samples on the direct microscopic examination of peripheral blood smears, which confirms the low sensitivity of this method in detecting *E. canis* in peripheral blood. Although autologous cell cultures are useful in propagating leucocytes for the diagnosis of *Anaplasma/Ehrlichia*, the attrition rates experienced in this study were moderately high, with successful culture results obtained for 58.8% of samples. It is important that a sterile serum is used and aseptic techniques are maintained. Given that the identical aseptic procedures were used for all samples, attrition was not likely due to a lack of sterility. However, it was observed that dogs with a concurrent bacteraemia may have contributed to bacterial multiplication in vitro and subsequent cell death. Other factors which influenced the viability of cell cultures included obtaining an insufficient starting volume of plasma and leucocytes. Some of the dogs were dehydrated, resulting in insufficient plasma volumes being recovered from these animals. Obtaining a sufficient volume of blood for the culture of *Anaplasma/Ehrlichia* is a known constraint. In order to obtain adequate canine cell culture lines, it is suggested that up to 5 to 30 mL of blood should be collected [5,6,7]. Collecting large volumes of blood, therefore, would be a constraint in a clinical setting. Short-term culture has been used by others for the diagnosis and treatment of canine ehrlichiosis in studies on naturally occurring *E. canis* [8,9]. Severe pancytopaenia may have also contributed to the insufficient recovery of leucocytes for propagation. Three samples that were considered positive on culture were negative by RLB. False positive results may have occurred if lymphoglandular bodies and phagocytosed nuclear materials were confused with morulae [5,7]. All of the three dogs that had positive culture and negative RLB results were from group 1. On the physical examination of these three dogs, all had normal body temperatures. However, ecchymotic haemorrhages were observed on the skin of one dog, and the other two appeared in good condition with no noticeable clinical signs of disease.

As the RLB involved first extraction and then deoxyribonucleic acid (DNA) amplification, false negative results may have been obtained if polymerase chain reaction (PCR) inhibitors were present as well as a low number of *E. canis* bacteria present, hence few infected cells present in the sample used to extract DNA. No other amplifiable gene such as canine Glyceraldehyde 3-phosphate dehydrogenase (GAPDH) (house-keeping gene) was screened for. Therefore, it is unknown if there was enough amplifiable DNA or if PCR inhibitors were present in the samples, both of which could lead to false negative results.

This study also reinforced that a haemogram for *E. canis*-positive dogs may be non-specific as only one *E. canis*-positive dog that was clinically ill with a positive cell culture and RLB result had a classical haemogram of pancytopaenia. As the sensitivity of RLB is higher than PCR, RLB can detect early infections resulting in earlier treatments and a better prognosis. Similarly, if the organism can grow then it is usually in the acute phase of the disease. Therefore, these two methods can be used to detect early *E. canis* infections. The small sample size of clinical cases was a limiting factor in this study; however, we were able to demonstrate that RLB can be used to confirm the presence of *E. canis* in clinically ill and apparently healthy dogs.

## 4. Materials and Methods

The Cannon and Roe (1982) formula [10] was used to estimate the minimum sample size needed to detect one case of the disease in free-roaming dogs. The minimum sample size needed to detect one positive dog using an estimated prevalence of *E. canis* of 14.0% [11], an estimated population size of 10,000 dogs, and a 95% confidence interval, was 20. Additionally, any dog that visited veterinary hospitals and clinics during the study period and met the inclusion criteria was included in the study.

Samples of 6 mL heparin and 3 mL EDTA blood were collected aseptically by sterile venepuncture from 28 dogs at the humane shelter. A physical examination including rectal temperature was performed on each dog, and the presence/absence of ticks was also recorded. Similarly, samples were submitted by veterinarians from dogs showing two or more clinical signs consistent with tick fever i.e., anorexia, lethargy, pyrexia, bleeding, and petechial and ecchymotic haemorrhages. These dogs were not treated for a tick-transmitted disease within the past month before blood collection. For each dog, a CBC was performed on the EDTA blood sample using an automated haemoanalyser (Sysmex K-4500 (Sysmex Co-operation, Kobe, Japan)). In addition, Giemsa-stained thin blood smears from all samples were examined microscopically for the characteristic inclusion bodies in leucocytes.

For the processing of samples for autologous cell cultures, heparinised samples were allowed to stand vertically at room temperature for 1 h for erythrocyte sedimentation. After sedimentation of erythrocytes, 2 mL of plasma and leucocytes were collected aseptically into Leighton tubes (Corning Inc., Corning, NY, USA). The tubes were incubated at 37 °C for 48 h under atmospheric conditions after which the plasma was discarded and the tubes washed with Dulbecco’s Modified Eagle’s medium without antibiotics (Sigma-Aldrich, Atlanta Georgia, GA, USA). Two millilitres of Dulbecco’s Modified Eagle’s medium without antibiotics, containing 20% canine homologous serum (Biomeda Corporation, Foster City, CA, USA), was then added to each tube and incubated as previously described. The tubes were subsequently fed with the same medium after 24 h. At 48 h post-incubation, the cover slips were removed, stained with Wright-Giemsa, and mounted with Canada Balsam^®^. They were subsequently microscopically examined under oil immersion at ×1000 to detect the presence of inclusion bodies in mononuclear cells. The person interpreting the slides was not aware of the clinical signs of the dog and its associated RLB results.

DNA was extracted from 100 μL heparinised blood using the DNeasy blood and tissue kit (Qiagen Maryland, Germantown, MD, USA) according to the manufacturer’s instructions. Extracted DNA was then stored at −20 °C in an elution buffer (AE) until required for analysis. RLB was performed as previously described by Bekker et al. (2002) for the 16S rRNA gene [12]. The positive control that was used is *E. canis* DNA that was previously confirmed by sequencing. The negative control was a negative extraction control using PCR grade water. Positive *E. canis* DNA obtained from this study are available on GenBank^TM^ EMBL and DDBJ databases under the accession numbers KY010672 to KY010674.

In the sample population, anaemia was classified as a haematocrit value < 0.35 L/L and thrombocytopaenia for platelet counts <200 × 10^9^ L/L in samples without platelet aggregates. Standard reference intervals for haematological parameters were used [13].

Data was analysed using SPSS version 20. The kappa statistic was calculated to determine the observed proportional agreement between culture and RLB results for dogs sampled from the Humane Society (group 1) and suspect cases (group 2). Statistical significance was set at a *p*-value of ≤ 0.05.

## 5. Conclusions

This study shows that both reverse line blot hybridisation and autologous cell cultures can be used to detect *E. canis* in subclinical and clinical cases of the disease. A definitive diagnosis of *E. canis* is best achieved by a combination of clinical signs, positive autologous cell culture, and reverse line blot hybridisation results.

## Figures and Tables

**Table 1:** RLB and autologous cell culture results for dogs in group 1( Humane Society).

Sample ID	Gender	Haematocrit (HCT, L/L)	Cell Culture	RLB
		(Ref. Range 0.37–0.55)	Result	Result ^1^
1	Female	0.43	positive	negative
2	Female	0.56	no result	negative
3	Female	0.44	no result	negative
4	Female	0.21	positive	A/Eall, *E. canis*
5	Female	0.38	positive	A/Eall, *E. canis*
6	Male	0.36	negative	negative
7	Male	0.49	positive	negative
8	Male	0.48	no result	negative
9	Female	0.43	no result	negative
10	Female	0.46	no result	negative
11	Male	0.46	negative	A/Eall
12	Female	0.47	negative	negative
13	Male	0.38	no result	negative
14	Male	0.51	no result	negative
15	Male	0.31	no result	negative
16	Male	0.37	negative	negative
17	Female	0.40	no result	negative
18	Male	0.42	no result	negative
19	Male	0.37	positive	negative
20	Male	0.41	no result	A/Eall, *E. canis*
21	Female	0.46	negative	negative
22	Female	0.37	negative	negative
23	Male	0.52	positive	A/Eall
24	Male	0.34	negative	negative
25	Male	0.36	negative	negative
26	Female	0.38	negative	negative
27	Female	0.42	negative	negative
28	Male	0.60	no result	negative

^1^ A/E all = positive for *Anaplasma/Ehrlichia* genera.

**Table 2:** RLB and autologous cell culture results for dogs in group 2 (suspect positive cases).

Sample ID	Gender	Haematocrit (HCT, L/L)	Cell Culture	RLB
		(Ref. Range 0.37–0.55)	Result	Result ^1^
1	Female	0.28	no result	negative
2	Female	0.26	positive	A/Eall, *E. canis*
3	Female	0.34	negative	negative
4	Female	0.44	negative	negative
5	Female	0.26	positive	A/Eall, *E. canis*
6	Female	0.34	no result	A/Eall, *E. canis*

**^1^** A/E all = positive for *Anaplasma/Ehrlichia* genera.

**Table 3:** A comparison of the RLB and culture results for *Anaplasma/Ehrlichia* infection.

Comparison of RLB and Culture Results for *Anaplasma/Ehrlichia* ^1^. (Kappa = 0.565, *p* = 0.01)			
	Culture	Culture	Total
	Positive	Negative	
RLB positive	5.0	1.0	6.0
RLB negative	3.0	11.0	14.0
Total	8.0	12.0	20.0

^1^ A total of 11 samples were RLB-negative with no culture result and 3 samples were RLB-positive with no culture result.

## Data Availability

All of the data generated in this study is presented in this manuscript.
